# The fatty liver index and risk of incident venous thromboembolism: the Tromsø Study

**DOI:** 10.1016/j.rpth.2024.102447

**Published:** 2024-05-20

**Authors:** Luuk J.J. Scheres, Sigrid K. Brækkan, Judith P.L. Verlaan, Suzanne C. Cannegieter, John-Bjarne Hansen, Vânia M. Morelli

**Affiliations:** 1 Department of Clinical Epidemiology, Leiden University Medical Center, Leiden, the Netherlands; 2 Department of Internal Medicine, Radboud University Medical Center, Nijmegen, the Netherlands; 3 Thrombosis Research Group, Department of Clinical Medicine, UiT - The Arctic University of Norway, Tromsø, Norway; 4 Thrombosis Research Center, Division of Internal Medicine, University Hospital of North Norway, Tromsø, Norway

**Keywords:** epidemiology, fatty liver, obesity, venous thromboembolism, venous thrombosis

## Abstract

**Background:**

For the relationship between obesity and venous thromboembolism (VTE), nonalcoholic fatty liver disease (recently termed metabolic dysfunction–associated steatotic liver disease) is of interest given the hepatic role in hemostasis.

**Objectives:**

We aimed to assess the association between the fatty liver index (FLI), as a proxy for nonalcoholic fatty liver disease, and VTE risk in a population-based cohort.

**Methods:**

Data from the Tromsø 4 (1994-1995) and 6 (2007-2008) surveys were used to calculate the FLI in 9870 participants. All VTEs were recorded up to December 31, 2020. We used Cox regression to estimate hazard ratios for VTE with 95% CIs by FLI groups defined according to clinical cut-offs (<30, 30-59, and ≥60). Because waist circumference and body mass index (BMI) are main determinants for FLI calculation, we assessed the potential contribution of FLI to VTE risk beyond these body fat measures.

**Results:**

During a median follow-up of 13.1 years, 507 incident VTEs occurred. Compared with the reference group (FLI < 30), the hazard ratios for VTE were 1.5 (95% CI, 1.1-1.9) and 1.8 (95% CI, 1.4-2.3) for the FLI 30-59 and ≥60 groups, respectively, in models adjusted for age, sex, alcohol intake, educational level, and physical activity. The association of FLI with VTE was no longer observed, with risk estimates close to unity, when participants were stratified by clinical categories of waist circumference and BMI.

**Conclusion:**

Higher values of the FLI were associated with a higher VTE risk. This association was explained by waist circumference and BMI, which reflect excessive body fat deposition and are determinants of the FLI.

## Essentials

• Data on nonalcoholic fatty liver disease and venous thromboembolism (VTE) are scarce.
• In the Tromsø Study, the fatty liver index (FLI) as a nonalcoholic fatty liver disease proxy was used to evaluate VTE risk.
• Higher values of the FLI were associated with a higher VTE risk.
• The findings were explained by the FLI determinants waist circumference and body mass index.

## 1 Introduction

Obesity, with its high and continuously rising prevalence worldwide [1], is one of the most important modifiable risk factors for both venous thromboembolism (VTE) and arterial thrombotic events [[2], [3], [4]]. However, obesity is typically defined based on crude measures, such as the body mass index (BMI), which does not take into account different clinical phenotypes with respect to specific locations of body fat distribution (eg, abdominal or visceral) and proportion of liver fat deposition [5]. Excessive liver fat deposition in the absence of excessive alcohol intake, often termed nonalcoholic fatty liver disease (NAFLD), is a common condition affecting up to 30% of the general population, with estimates increasing with higher BMI [6]. Recently, metabolic dysfunction–associated steatotic liver disease (MASLD) was proposed as a new nomenclature to address this condition, which underscores hepatic steatosis as a key pathophysiologic component while assigning the metabolic basis for the liver disease [7]. It has been reported that the risk of arterial cardiovascular events is increased in persons with NAFLD [8]. In contrast, less data are available on the association between NAFLD and risk of VTE. The role of liver fat deposition could be of particular interest in this context, since the liver is the main site of production of coagulation and anticoagulation factors and their respective higher and lower levels are associated with risk of VTE [9,10]. In line with this, hepatic triglyceride content, a measure of the degree of excessive liver fat deposition, was associated with higher levels of several coagulation factors [11]. In terms of clinical outcomes, available data are limited to a relatively small case-control study (138 VTE cases, 276 controls), involving only patients with unprovoked VTE, where the prevalence of NAFLD was approximately 2-fold higher in cases than in controls [12]. Understanding the association between NAFLD and VTE risk could provide valuable insights for enhancing risk stratification beyond BMI alone. In addition, it may provide more direction for the development of new preventive and therapeutic approaches for VTE especially in the advent of new therapies being developed for NAFLD [13].

In the current study, we aimed to investigate the potential association between NAFLD and the risk of VTE. The clinical diagnosis of NAFLD is typically based on imaging studies and invasive liver biopsies in some cases [14]. For epidemiologic research purposes, in which invasive or extensive imaging studies are less appealing or even impractical, several diagnostic scores have been developed. Therefore, we used the fatty liver index [15], an externally validated score (calculated based on BMI, waist circumference [WC], triglycerides, and gamma-glutamyl transferase [GGT]), as a proxy to assess the association between NAFLD and VTE risk. In addition, we aimed to evaluate whether BMI and WC, which are main components of the fatty liver index and established risk factors for VTE [4,16,17], could explain a potential association between the fatty liver index and VTE risk.

## 2 Methods

### 2.1 Study population and design

We used data from the Tromsø Study, which is a Norwegian population–based cohort study with repeated health surveys of the inhabitants of Tromsø, Norway [18]. For the present study, based on the availability of data required to calculate the fatty liver index, the study population was derived from the fourth and sixth surveys. In the fourth survey (1994-1995), all inhabitants of the Tromsø municipality aged ≥25 years were invited to participate and a total of 27,158 persons (77%) participated. Of these, 7965 were invited to a second more extensive survey (ie, all inhabitants aged 55-74 years and 5%-10% random samples from the age groups 25-54 years and 75-85 years). In the sixth survey (2007-2008), 12,984 persons aged 30 to 87 years were included (66% of those invited). Data required for the fatty liver index was available in the Tromsø 4 (second visit) and Tromsø 6 surveys. Participants with a history of VTE (*n* = 126), self-reported history of liver disease (*n* = 218), and self-reported use of >2 units of alcohol per day (*n* = 397) were excluded, leaving 9870 unique individuals in the study. Participants were followed from the date of inclusion in the study until occurrence of an incident VTE, migration, death, or end of follow-up. The study was approved by the Regional Committee for Medical and Health Research Ethics, and all participants signed an informed consent form prior to inclusion.

### 2.2 Baseline assessment of fatty liver index

In this study we used the fatty liver index as a proxy for NAFLD [15]. The fatty liver index, which ranges from 0 to 100, was calculated as described before based on the variables BMI, WC, and triglyceride and GGT levels [15] according to the following equation:

$$\text{Fatty}\;\text{liver}\;\text{index}=\left(e^{0.953\times \text{loge}\left(\text{triglycerides}\right)+0.139\times \text{BMI}+0.718\times \text{loge}\left(\text{GGT}\right)+0.053\times \text{waist}\;\text{circumference}\;-\;15.745}\right)\;/\;\left(1\;+\;e^{0.953\times \text{loge}\left(\text{triglycerides}\right)+0.139\times \text{BMI}+0.718\times \text{loge}\left(\text{GGT}\right)+0.053\times \text{waist}\;\text{circumference}\;-\;15.745}\right)\times 100\text{.}$$

In the development study, the fatty liver index of <30 ruled out excessive fatty liver deposition and a value of ≥60 was proposed to rule in fatty liver deposition [15]. The score performed well in external validation studies in Western European populations for the prediction of NAFLD as identified by ultrasonography [19] and proton magnetic resonance spectroscopy [20].

In the Tromsø Study, height and weight were measured with participants wearing light clothes and no shoes, and BMI was calculated as weight in kilograms divided by the square of height in meters (kg/m^2^). The WC was measured in centimeters at the umbilical line. Nonfasting blood samples were used for the assessment of serum triglycerides and GGT by standard methods at the University Hospital of North Norway.

### 2.3 Other baseline measurements

Baseline information on alcohol intake, history of cardiovascular disease (CVD), physical activity, and educational status was obtained by standardized and validated questionnaires. Based on the information in the questionnaire, self-reported daily average alcohol use was grouped into teetotalers, 0 to 1 unit per day, and >1 to 2 units per day. A history of CVD was defined as either self-reported chest angina, myocardial infarction, or stroke. Physical activity and educational status were grouped into >1 hour per week of physical activity (yes/no) and college/university level (yes/no), respectively. Some values were missing for alcohol use (17.3%), physical activity (5.5%), history of CVD (1.0%), and educational status (0.4%).

### 2.4 Identification of VTE during follow-up

The ascertainment of incident VTE events during follow-up was described in detail previously [18,21,22]. In short, incident VTE events among study participants were recorded from the date of enrolment in Tromsø 4 and 6 through the end of follow-up (December 31, 2020). All first-life time VTE events were identified by crosschecking the hospital’s discharge registry, the radiology procedure registry, and autopsy registry at the University Hospital of North Norway, which is the only hospital in the region providing diagnostics and care of VTE patients. Following this, trained health care providers adjudicated VTE events by an extensive review of the medical records. A VTE was confirmed if signs and symptoms of deep vein thrombosis or pulmonary embolism were combined with objective confirmation (by radiological procedures) and treatment initiation (unless contraindications were specified). For VTE outcomes based on the autopsy register, a VTE event was adjudicated if the record of the autopsy designated VTE as the cause of death or a significant condition associated with the death.

VTE episodes were categorized into unprovoked or provoked depending on the presence of specific risk factors closely preceding the VTE. Provoking risk factors include major surgery, trauma, acute medical conditions (ie, myocardial infarction, ischemic stroke, and major infectious disease) within 8 weeks before the events, active cancer at the time of VTE diagnosis, immobilization (bed rest > 3 days or confinement to wheelchair within the last 8 weeks, or long-distance travel of ≥4 hours within the last 14 days), or other risk factors explicitly described by the treating physicians in the medical records (such as intravascular catheters).

### 2.5 Statistical analysis

Statistical analysis was carried out with SPSS (version 26.0). Means (± SD) and proportions of baseline characteristics were calculated using descriptive statistics. Follow-up time started at inclusion in the first survey with complete information to calculate the fatty liver index, and ended at time of incident VTE, migration from Tromsø, death, or end of follow-up (December 31, 2020), whichever occurred first. Incidence rates with 95% CIs (based on a Poisson distribution) were calculated by dividing the number of incident VTEs by the total observation time and expressed as number of events per 1000 person-years. As the main analyses, we used Cox proportional hazard models to estimate hazard ratios (HRs) and corresponding 95% CIs for VTE according to the previously defined clinical cut-off values for the fatty liver index (<30 [reference group], 30-59, and ≥60). Additionally, we performed the analyses based on quartiles of the fatty liver index determined in the study population, with the lowest quartile as the reference group. The confounders age, sex, alcohol intake, education status, and physical activity were added to the models in a stepwise manner. When values for these variables were missing, complete case analyses were performed. Subgroup analyses were performed separately for unprovoked and provoked VTE. Moreover, as WC has sex-specific cut-off values [23], which are not taken into account in the fatty liver index, we further carried out subgroup analysis in men and women separately using the clinical cut-offs of the fatty liver index. For sensitivity purposes, we performed an analysis for overall VTE excluding persons with a baseline self-reported history of CVD.

Finally, as BMI and WC are components of the score and established VTE risk factors [4,16,17], we performed analyses to evaluate whether the fatty liver index had the potential to contribute to VTE risk beyond BMI or WC. To this end, we evaluated the risk of VTE according to clinical cut-off values of the fatty liver index (<30, 30-59, and ≥60) within strata of clinical categories of WC and BMI defined by the World Health Organization (WC category 1: men, ≤94 cm; women, ≤80 cm; WC category 2: men, >94-102 cm; women, >80-88 cm; WC category 3: men, >102 cm; women, >88 cm; BMI < 25 kg/m^2^, 25-30 kg/m^2^, and ≥30 kg/m^2^) [23,24]. In each stratum of WC (ie, categories 1, 2, and 3) and BMI (ie, <25, 25-30, and ≥30 kg/m^2^), a fatty liver index of <30 served as the reference.

## 3 Results

The baseline clinical characteristics of the 9870 participants included in the study according to clinical cut-off values of the fatty liver index are depicted in Table 1. Overall, the mean age and the distribution of alcohol intake, educational status, and physical activity were similar across groups of fatty liver index. Notable differences among the groups concerned the distribution of sex and history of arterial CVD. The proportion of women decreased from 68.1% in fatty liver index < 30 group to 38.1% in the group with a fatty liver index of ≥60. The percentage of persons with a self-reported history of arterial CVD was lowest in the fatty liver index < 30 group and highest in the group with a fatty liver index of ≥60. As expected, the mean BMI, WC, and triglyceride and GGT levels increased with an increasing fatty liver index.

**Table 1:** Baseline clinical characteristics of the study participants (*n* = 9870) by clinical cut-off values of the fatty liver index.

	FLI < 30	FLI: 30 to 59	FLI ≥ 60
Total, *n*	4063	2870	2937
Age (y), mean ± SD	54.5 ± 13.0	56.3 ± 12.0	54.8 ± 12.2
Sex (women), *n* (%)	2768 (68.1%)	1265 (44.1%)	1118 (38.1%)
Physical activity^a^ (>1 h per wk), *n* (%)	1436 (35.3%)	971 (33.8%)	889 (30.3%)
Educational status^a^ (college or university), *n* (%)	1229 (30.2%)	752 (26.2%)	828 (28.3%)
Alcohol intake^a^			
Teetotaler, *n* (%)	697 (21.3%)	531 (22.4%)	509 (20.2%)
0 to 1 units per d, *n* (%)	2384 (72.9%)	1672 (70.5%)	1775 (70.6%)
>1 to 2 units per d, *n* (%)	190 (5.8%)	170 (7.2%)	231 (9.2%)
History of CVD^a^, *n* (%)	315 (7.8%)	377 (13.1%)	480 (16.6%)
Variables of the fatty liver index			
BMI (kg/m^2^), mean ± SD	23.1 ± 2.4	26.1 ± 2.4	29.4 ± 3.8
Waist circumference (cm), mean ± SD	81.7 ± 7.2	93.1 ± 5.9	104.0 ± 8.7
Triglycerides (mg·dL^−1^), mean ± SD	1.0 ± 0.4	1.5 ± 0.7	2.2 ± 1.2
GGT (U·L^−1^), mean ± SD	18.8 ± 11.3	26.7 ± 19.3	45.2 ± 52.2

BMI, body mass index; CVD, cardiovascular disease (history of myocardial infarction, stroke, or angina); GGT, gamma-glutamyl transferase; FLI, fatty liver index.

a Missing values for the variables: alcohol use (17.3%), physical activity (5.5%), history of CVD (1.0%), and educational status (0.4%).

During a median of 13.1 (IQR, 12.7-24.5) years of follow-up, a total of 507 incident VTEs occurred at an overall incidence rate of 3.2 (95% CI, 3.0-3.5) per 1000 person-years. The incidence rates of VTE and the corresponding HRs for each of the fatty liver index groups based on the clinical cut-off values are shown in Table 2. In all 3 models, the estimated HRs increased across the fatty liver index categories, with no substantial change in risk estimates upon adjustment for potential confounders. In the fully adjusted model, compared with the HRs for the reference group, the HRs for VTE were 1.5 (95% CI, 1.1-1.9) and 1.8 (95% CI, 1.4-2.3) for the fatty liver index 30-59 and ≥60 groups, respectively. A similar pattern of the results was observed when separating the analyses by provoked or unprovoked VTE (Table 2). In the groups with a fatty liver index of ≥60, the HRs were 1.6 (95% CI, 1.1-2.2) for provoked VTE and 2.0 (95% CI, 1.3-3.1) for unprovoked VTE compared with those of the reference group.

**Table 2:** Incidence rates and hazard ratios of venous thromboembolism by clinical cut-off values of the fatty liver index.

	Person-y, *n*	VTE events, *n*	Crude IR (95% CI)^a^	HR^b^ (95% CI)	HR^c^ (95% CI)	HR^d^ (95% CI)
Overall VTE						
FLI < 30	67,931	169	2.5 (2.1-2.9)	1 (ref)	1 (ref)	1 (ref)
FLI: 30 to 59	45,980	170	3.7 (3.2-4.3)	1.4 (1.1-1.7)	1.4 (1.1-1.9)	1.5 (1.1-1.9)
FLI ≥ 60	43,114	168	3.9 (3.3-4.5)	1.6 (1.3-2.0)	1.7 (1.3-2.2)	1.8 (1.4-2.3)
Provoked VTE						
FLI < 30	67,931	106	1.6 (1.3-1.9)	1 (ref)	1 (ref)	1 (ref)
FLI: 30 to 59	45,980	101	2.2 (1.8-2.7)	1.3 (1.0-1.7)	1.3 (0.9-1.8)	1.3 (1.0-1.9)
FLI ≥ 60	43,114	99	2.3 (1.9-2.8)	1.5 (1.1-2.0)	1.5 (1.1-2.0)	1.6 (1.1-2.2)
Unprovoked VTE						
FLI < 30	67,931	63	0.9 (0.7-1.2)	1 (ref)	1 (ref)	1 (ref)
FLI: 30 to 59	45,980	69	1.5 (1.2-1.9)	1.5 (1.1-2.2)	1.7 (1.1-2.6)	1.7 (1.1-2.6)
FLI: ≥60	43,114	69	1.6 (1.2-2.0)	1.8 (1.3-2.6)	2.1 (1.4-3.1)	2.0 (1.3-3.1)

FLI, fatty liver index; HR, hazard ratio; IR, incidence rate; ref, reference; VTE, venous thromboembolism.

a IRs per 1000 person-years.

b Adjusted for sex and age.

c Adjusted for sex, age, and alcohol intake.

d Adjusted for sex, age, alcohol intake, educational level, and physical activity.

**Figure 1:**
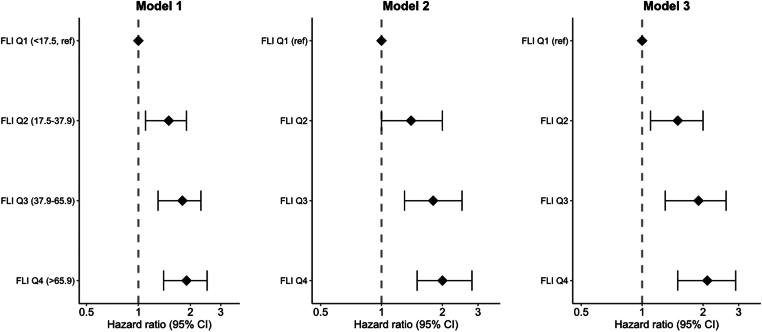
Quartiles of the FLI and risk of incident VTE. Hazard ratios with 95% CIs for incident VTE are shown by quartiles (Q1 to Q4) of the FLI in the study population, with the first quartile (Q1) as reference. The models are adjusted for age and sex (model 1), + alcohol intake (model 2), and + educational status and physical activity (model 3, full model). CI, confidence interval; FLI, fatty liver index; VTE, venous thromboembolism.

A stepwise increase in VTE risk was also observed for the analyses by quartiles of the fatty liver index (Figure 1), with a HR of 2.1 (95% CI, 1.5-2.9) for the fourth quartile compared with that for the first quartile (reference category) in the fully adjusted model.

**Figure 2:**
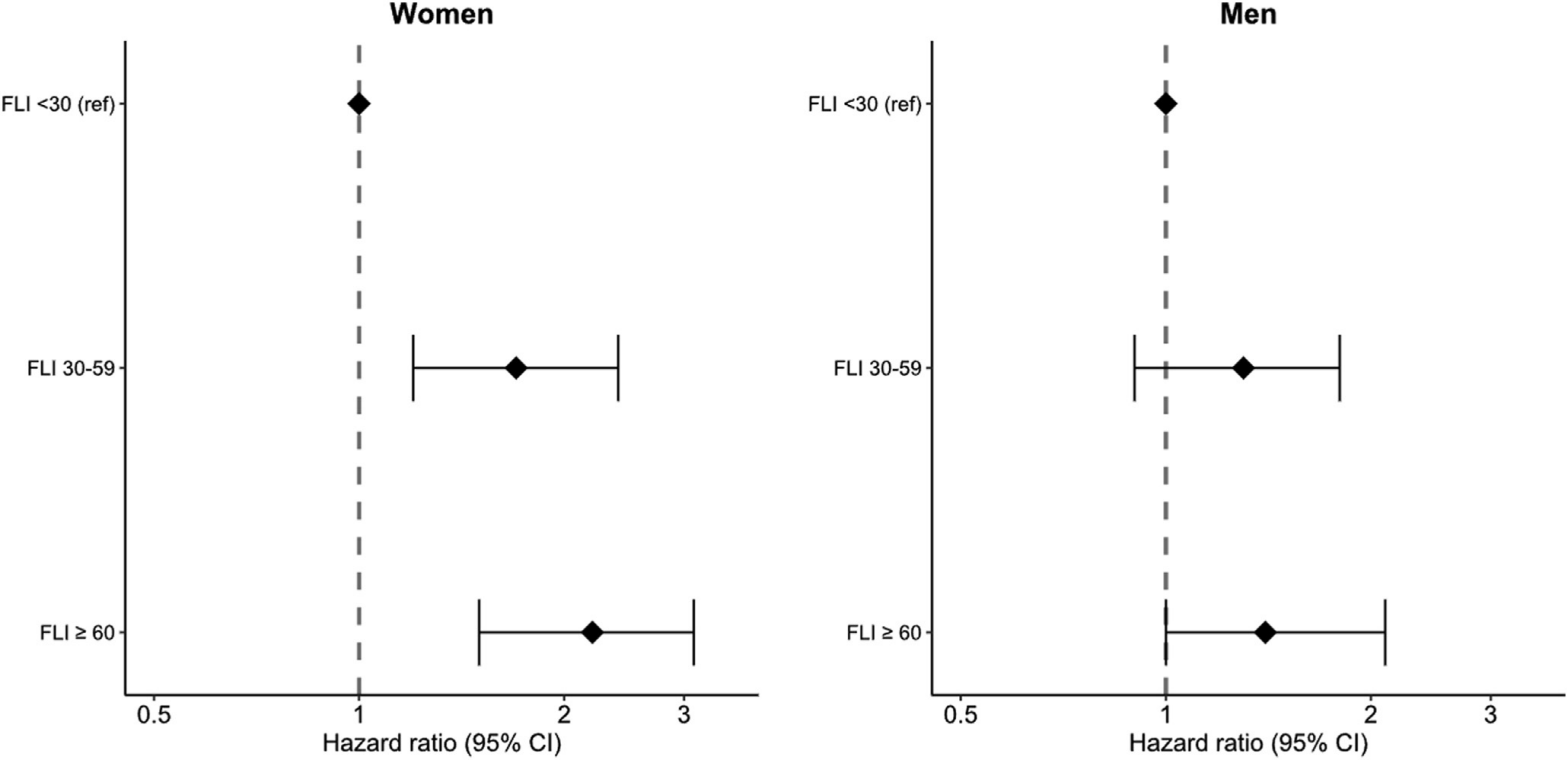
Risk of VTE in women and men by clinical cut-off categories of FLI. Hazard ratios with 95% CIs for incident VTE are shown separately for women and men by clinical categories of FLI, with the FLI of <30 as reference. HRs are adjusted for age, alcohol intake, educational status, and physical activity. CI, confidence interval; FLI, fatty liver index; VTE, venous thromboembolism.

The results for the separate analyses among women and men based on the clinical cut-off values of the fatty liver index are shown in Figure 2, where the risk estimates of the fully adjusted model are depicted. Compared with the reference group, the increased risk for VTE in the fatty liver index group ≥ 60 was more pronounced in women (HR, 2.2; 95% CI, 1.5-3.1) than in men (HR, 1.4; 95% CI, 1.0-2.1). The results of the sensitivity analyses, in which persons with a self-reported history of arterial CVD at baseline were excluded, did also not differ from the main results (Supplementary Figure S1).

**Figure 3:**
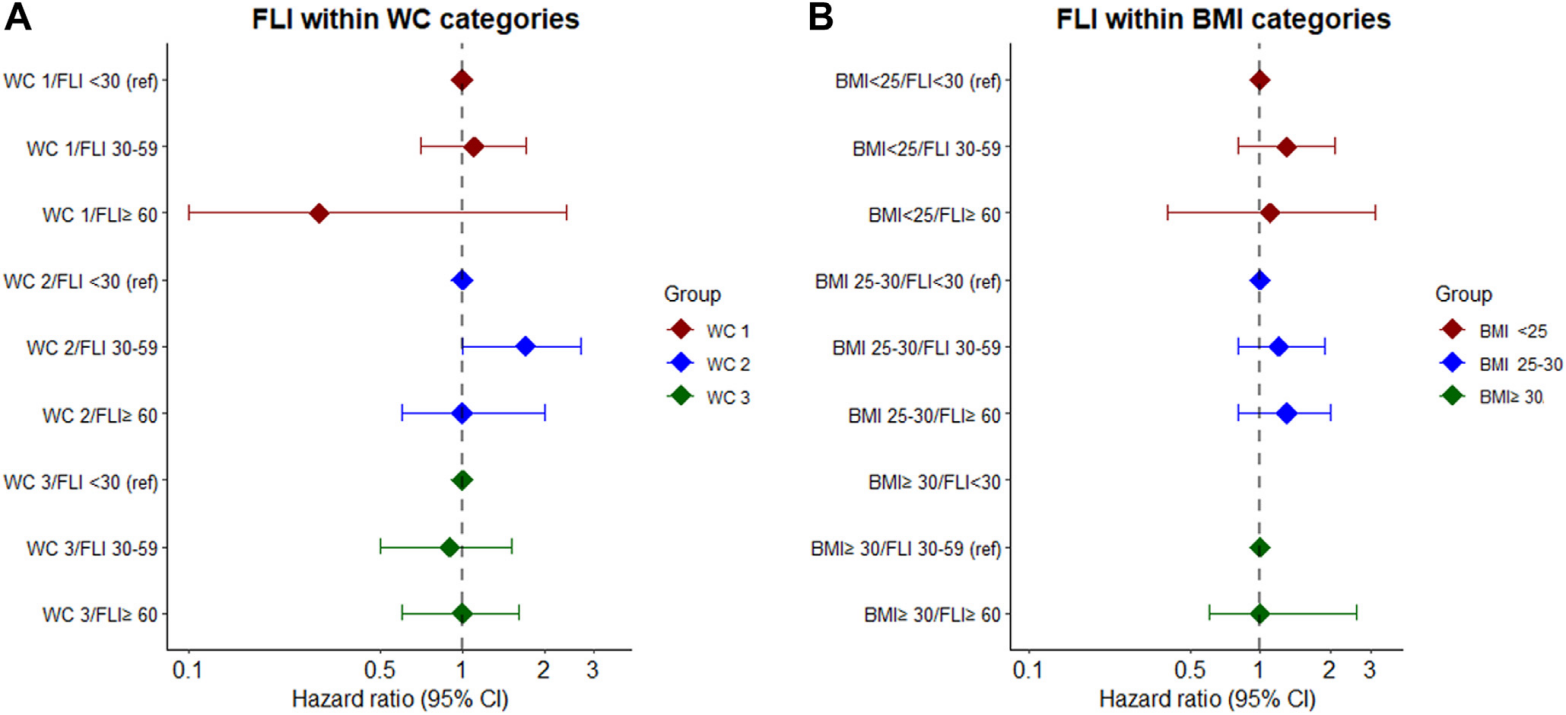
Risk of VTE by clinical categories of FLI within groups of (A) WC and (B) BMI. Hazard ratios with 95% CIs for incident VTE are shown by clinical categories of the FLI with (A) groups of WC and (B) groups of BMI, with the FLI of <30 as reference. The results of model 3 (full model), including age, sex, alcohol intake, educational status, and physical activity are shown. For panel A on the left, the WC categories concern the World Health Organization definitions: WC category 1 (red), ≤94 cm for men and ≤80 cm for women; WC category 2 (blue), >94 to 102 cm for men and >80 to 88 cm for women; and WC category 3 (green), >102 cm for men and >88 cm for women. For panel B on the right, the BMI categories concern the World Health Organization definitions: category 1, BMI < 25 kg/m^2^ (red); category 2, BMI = 25 to 30 kg/m^2^ (blue); and category 3, BMI ≥ 30 kg/m^2^ (green). In the BMI ≥ 30/FLI < 30 group there were no events, hence no estimate is shown and the subsequent group (ie, BMI ≥ 30/FLI, 30-59) is used as the reference. BMI, body mass index; CI, confidence interval; FLI, fatty liver index; HR, hazard ratio; VTE, venous thromboembolism; WC, waist circumference.

As previously noted, WC and BMI are main determinants of the fatty liver index and are also independently associated with VTE [4,16,17]. To further assess whether and to what extent the association between the fatty liver index and VTE risk could be explained by these determinants, we assessed the risk of VTE within each stratum of WC and BMI. As depicted in the fully adjusted model in Figure 3, within each stratum of WC (Figure 3A) or BMI (Figure 3B), the fatty liver index was not associated with VTE risk, with most of the risk estimates close to unity (detailed tables are available as Supplementary Tables S1 and S2).

## 4 Discussion

In this population–based cohort study among nearly 10,000 persons with a median follow-up of 13 years, we assessed the association between the fatty liver index as a proxy for NAFLD and the risk of incident VTE. Increasing values of the fatty liver index based on clinical cut-off groups were associated with an up to 1.8-fold increased risk of VTE. These findings were similar for the analyses based on quartiles of the fatty liver index and when excluding persons with a history of arterial CVD. The results were slightly more prominent among women and for unprovoked VTE events. Most importantly, the association between fatty liver index and VTE seemed to be essentially explained by WC and BMI, as the association disappeared when we stratified for these measurements of body fat deposition.

The interpretation of our results is not straightforward because of the overlapping role of WC and BMI as determinants of the fatty liver index and as individual risk factors for VTE. As the fatty liver index is well validated, the prevalence of NAFLD is likely high among the persons in our study with a fatty liver index of ≥60. This suggests that NAFLD indeed is associated with an increased VTE risk, as observed in the current study. However, provided that WC and BMI are main determinants of the score, a high fatty liver index also likely reflects a high proportion of other localizations of body fat deposition such as abdominal fat deposition and obesity. Hence, WC, BMI, and the fatty liver index can be considered related proxy measurements of excessive body fat deposition and obesity, in which their individual associations with VTE risk are likely mediated by common biological pathways. In the current study, we cannot further distinguish the actual contribution of NAFLD to the observed VTE risk beyond the pathways that are represented by WC and BMI. As previously mentioned, other studies investigating the risk of VTE among persons with NAFLD are limited. In the case-control study by Di Minno et al. [12], the prevalence of NAFLD among those with unprovoked VTE was higher than in age-, sex-, and BMI-matched controls: in 112/138 cases (81%) and in 84/276 controls (30%), which does suggest a role of NAFLD in VTE risk beyond BMI. Our findings are in line with a recently published cohort study (*n* = 472,212) in a Korean population, in which the fatty liver index was also employed as a proxy for NAFLD. In this study, similar to our observations, increasing values of the fatty liver index were also associated with an increased VTE risk, with a HR of 1.45 (95% CI, 1.30-1.62) in the fourth quartile compared with that in the first quartile in the fully adjusted model [25]. However, the VTE diagnosis was based solely on the International Classification of Disease codes, and the potential of the fatty liver index to contribute to the VTE risk beyond BMI and WC was not assessed [25].

Overall, our findings suggest that any association of the fatty liver index with VTE risk is likely due to measures related to excessive body fat deposition and obesity and that to determine whether NAFLD is associated with VTE risk independent of obesity would require in principle a different definition of NAFLD rather than the fatty liver index. For instance, imaging studies or specific biomarkers of fatty liver would be useful to further evaluate the risk of VTE in NAFLD, independent of body fat deposition in other localizations. However, one of the key questions that remain open is whether the effect of NAFLD can be separated from that of excessive body fat deposition in other localizations, or whether it should be considered part of the overarching obesity spectrum. Further investigation of this association is of relevance, as the prevalence of NAFLD is expected to increase with the global increase of obesity [6,26]. Moreover, currently numerous therapies for NAFLD are under investigation [13]. If there is indeed a causal relationship between NAFLD and VTE, these new therapies may also be of interest in the context of VTE risk reduction. To answer these questions ideally, the risk of VTE should be studied by comparing discordant pairs with NAFLD assessment based on imaging studies: for example, persons with low BMI and NAFLD and persons with high BMI without NAFLD. However, this approach may be challenging in terms of feasibility as the number of persons within these subgroups is likely small. Moreover, risk estimates of VTE throughout the NAFLD spectrum (ie, from low-risk steatosis to high-risk steatohepatitis) could provide further insight. In summary, in light of our findings, the fatty liver index, or other diagnostic scores in which measurements (such as WC and BMI) of body fat deposition are used, may be impractical for further studies on VTE risk.

Strengths of the current study include its large sample size and number of objectively validated VTE events during follow-up. Information for the calculation of the fatty liver index was available for a large group of persons, and we were able to exclude persons with excessive alcohol use, increasing the likelihood that a high fatty liver index conveyed a high prevalence of NAFLD. Using a cohort design, we were able to establish a clear temporal sequence between exposure (fatty liver index) and outcome (VTE). Another strength that requires attention is that we evaluated whether and to what extent the association between fatty liver index and VTE was explained by its main determinants (ie, WC and BMI), which are also associated with VTE occurrence. The fatty liver index has been investigated in context of arterial cardiovascular outcomes, in which WC and BMI are also established risk factors [[27], [28], [29], [30], [31], [32]]. In these studies, however, the association between the fatty liver index and the outcome of interest was not assessed beyond WC and BMI as components of the fatty liver index like we did in the current study.

Several limitations also require attention when interpreting the results of the current study. First, as an initial step in the assessment of the risk of VTE in persons with NAFLD, we used the fatty liver index as an alternative to invasive or logistically challenging imaging procedures. Although the score is externally validated in Western European populations similar to the population in the Tromsø surveys, misclassification of the exposure cannot be ruled out. However, any potential misclassification would be most likely not different among participants with and without (ie, nondifferential) a future VTE, thereby introducing a possibility of underestimation of the observed association between the fatty liver index and VTE risk. Moreover, detailed information on ethnicity was not available and our findings reflect a Norwegian population, and the obtained results may not be universally generalizable and could vary across different populations. In addition, we were not able to investigate the severity of NAFLD throughout the disease spectrum. Furthermore, assessment of important variables, such as alcohol intake, relied on self-reported numbers, which are also prone to misclassification. As noted, recently new nomenclature has been proposed replacing NAFLD with MASLD [7]. Although there is substantial overlap between the old NAFLD and new MASLD definitions, the accuracy of fatty liver index has not been evaluated for this definition, which also includes at least 1 of 5 cardiometabolic risk factors [7]. Nonetheless, the impact of the new definition is likely minor, since in an analysis of the European LITMUS consortium, 98% of the existing registry cohort of persons with NAFLD would fulfill the new criteria for MASLD [7,33]. Finally, our analysis on the association between fatty liver index and VTE risk according to WC and BMI strata (Figure 3) was based on low numbers of VTE events in some subgroups, with an inherent limited statistical power.

In conclusion, higher values of the fatty liver index were associated with a higher risk of incident VTE. This association was explained by WC and BMI, which reflect excessive body fat deposition and are main determinants of the fatty liver index.
