# Supplementary material for: The fatty liver index and risk of incident venous thromboembolism: the Tromsø Study
Source: Res Pract Thromb Haemost. 2024 May 20;8(4):102447. doi: 10.1016/j.rpth.2024.102447 (PMC11215415; doi:10.1016/j.rpth.2024.102447)
Supplement: Supplementary Material [file mmc1.docx]

**Supplementary Figure S1**. Risk of venous thromboembolism (VTE) by clinical categories of the fatty liver index (FLI). Sensitivity analyses excluding persons with a personal history of cardiovascular disease (CVD) at baseline.


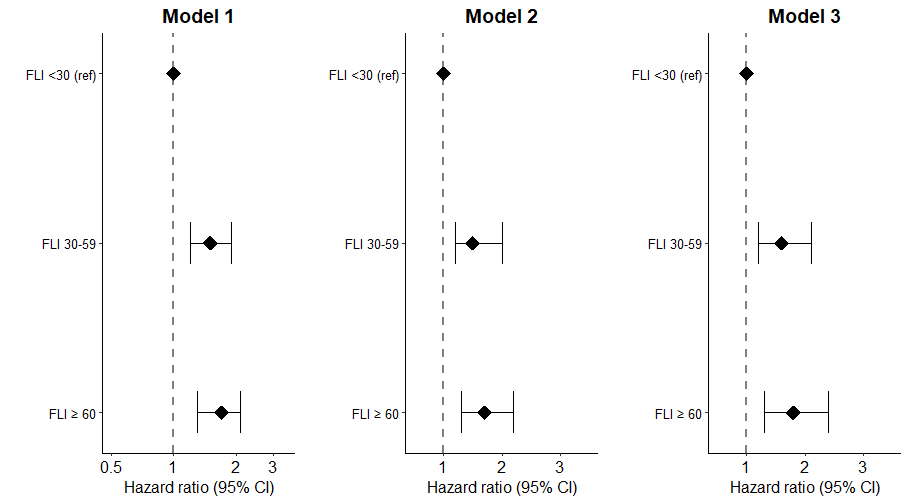


Hazard ratios with 95%CI (confidence intervals) for incident VTE are shown by clinical categories of the fatty liver index in the study population, the FLI <30 as reference. The models are adjusted for age and sex (model 1), + alcohol intake (model 2) and + educational status and physical activity (model 3, full model).

| **Supplementary Table S1. Risk of VTE by clinical cut off values of the FLI within groups of WC** | | | | | | |
| --- | --- | --- | --- | --- | --- | --- |
|  | Person-years, n | VTE Events, n | Crude IR (95% CI)^#^ | HR*  (95% CI) | HR✝  (95% CI) | HR‡  (95% CI) |
| Overall VTE | | | | | | |
| **WC category 1** | | | | | | |
| WC category 1/  FLI <30 | 46 698 | 105 | 2.2  (1.8-2.7) | 1.0  (ref) | 1.0  (ref) | 1.0  (ref) |
| WC category 1/  FLI 30-59 | 13 710 | 28 | 2.0  (1.4-3.0) | 0.9  (0.6-1.4) | 1.0  (0.6-1.6) | 1.1  (0.7-1.7) |
| WC category 1/  FLI ≥ 60 | 2 096 | 2 | 1.0  (0.2-3.4) | 0.5  (0.1-2.0) | 0.3  (0.1-2.3) | 0.3  (0.1-2.4) |
| **WC category 2** | | | | | | |
| WC category 2/  FLI <30 | 16 519 | 43 | 2.6  (1.9-3.5) | 1.0  (ref) | 1.0  (ref) | 1.0  (ref) |
| WC category 2/  FLI 30-59 | 16 111 | 65 | 4.0  (3.1-5.1) | 1.5  (1.0-2.2) | 1.6  (1.0-2.5) | 1.7  (1.0-2.7) |
| WC category 2/  FLI ≥ 60 | 9 415 | 22 | 2.3  (1.5-3.5) | 1.0  (0.6-1.7) | 1.0  (0.5-1.8) | 1.0  (0.6-2.0) |
| **WC category 3** | | | | | | |
| WC category 3/  FLI <30 | 4 714 | 21 | 4.5  (2.8-6.8) | 1.0  (ref) | 1.0  (ref) | 1.0  (ref) |
| WC category 3/  FLI 30-59 | 16 159 | 77 | 4.8  (3.8-6.0) | 0.9  (0.5-1.4) | 0.9  (0.5-1.5) | 0.9  (0.5-1.6) |
| WC category 3/  FLI ≥ 60 | 31 604 | 144 | 4.6  (3.8-5.4) | 0.9  (0.6-1.5) | 1.0  (0.6-1.6) | 1.0  (0.6-1.7) |

IR; incidence rate, HR; hazard ratio, CI; confidence interval, FLI; fatty liver index, VTE; venous thromboembolism, WC; World Health Organization waist circumference categories: category 1, ≤94 cm for men and ≤80cm for women. Category 2, >94 – 102 cm for men and >80 - 88 cm for women. Category 3, >102 cm for men and >88 cm for women.

^#^IRs per 1000 person-years

*Adjusted for sex and age

✝ Adjusted for sex, age and alcohol intake

‡ Adjusted for sex, age, alcohol intake, educational level and physical activity

| **Supplementary Table S2. Risk of VTE by clinical cut off values of the FLI within groups of BMI** | | | | | | |
| --- | --- | --- | --- | --- | --- | --- |
|  | Person-years, n | VTE Events, n | Crude IR (95% CI)^#^ | HR*  (95% CI) | HR✝  (95% CI) | HR‡  (95% CI) |
| Overall VTE | | | | | | |
| **BMI <25 kg/m2** | | | | | | |
| BMI <25 /  FLI <30 | 52 098 | 119 | 2.3  (1.9-2.7) | 1.0  (ref) | 1.0  (ref) | 1.0  (ref) |
| BMI <25 /  FLI 30-59 | 13 632 | 37 | 2.7  (1.9-3.7) | 1.2  (0.8-1.8) | 1.2  (0.8-1.9) | 1.3  (0.8-2.1) |
| BMI <25 /  FLI ≥ 60 | 3 326 | 5 | 1.5 (0.5-3.5) | 1.1  (0.4-2.6) | 1.2  (0.5-3.0) | 1.1  (0.4-3.1) |
| **BMI 25-30 kg/m2** | | | | | | |
| BMI 25-30 /  FLI <30 | 15 588 | 50 | 3.2  (2.4-4.2) | 1.0  (ref) | 1.0  (ref) | 1.0  (ref) |
| BMI 25-30 /  FLI 30-59 | 29 227 | 116 | 4.0  (3.3-4.8) | 1.2  (0.9-1.7) | 1.2  (0.8-1.8) | 1.2  (0.8-1.9) |
| BMI 25-30 /  FLI ≥ 60 | 22 343 | 80 | 3.6  (2.8-4.5) | 1.3  (0.9-1.9) | 1.2  (0.8-1.9) | 1.3  (0.8-2.0) |
| **BMI ≥ 30 kg/m2** | | | | | | |
| BMI ≥ 30 /  FLI <30 | 245 | 0 | - | - | - | - |
| BMI ≥ 30 /  FLI 30-59 | 3 122 | 17 | 5.5  (3.2-8.7) | 1.0  (ref) | 1.0  (ref) | 1.0  (ref) |
| BMI ≥ 30 /  FLI ≥ 60 | 17 445 | 83 | 4.8  (3.8-5.9) | 1.0  (0.6-1.8) | 1.3  (0.6-2.6) | 1.3  (0.6-2.6) |

IR; incidence rate, HR; hazard ratio, CI; confidence interval, FLI; fatty liver index, VTE; venous thromboembolism, BMI; body mass index

^#^IRs per 1000 person-years

*Adjusted for sex and age

✝ Adjusted for sex, age and alcohol intake

‡ Adjusted for sex, age, alcohol intake, educational level and physical activity
